# Fully automatic classification of automated breast ultrasound (ABUS) imaging according to BI-RADS using a deep convolutional neural network

**DOI:** 10.1007/s00330-022-08558-0

**Published:** 2022-02-11

**Authors:** Patryk Hejduk, Magda Marcon, Jan Unkelbach, Alexander Ciritsis, Cristina Rossi, Karol Borkowski, Andreas Boss

**Affiliations:** 1 Institute of Diagnostic and Interventional Radiology, University Hospital Zurich, Rämistr. 100, 8091 Zurich, Switzerland; 2 Department of Radiation Oncology, University Hospital Zurich, Rämistr. 100, 8091 Zurich, Switzerland

**Keywords:** Ultrasonography, Mammary, Machine learning, Breast neoplasms

## Abstract

**Purpose:**

The aim of this study was to develop and test a post-processing technique for detection and classification of lesions according to the BI-RADS atlas in automated breast ultrasound (ABUS) based on deep convolutional neural networks (dCNNs).

**Methods and materials:**

In this retrospective study, 645 ABUS datasets from 113 patients were included; 55 patients had lesions classified as high malignancy probability. Lesions were categorized in BI-RADS 2 (no suspicion of malignancy), BI-RADS 3 (probability of malignancy < 3%), and BI-RADS 4/5 (probability of malignancy > 3%). A deep convolutional neural network was trained after data augmentation with images of lesions and normal breast tissue, and a sliding-window approach for lesion detection was implemented. The algorithm was applied to a test dataset containing 128 images and performance was compared with readings of 2 experienced radiologists.

**Results:**

Results of calculations performed on single images showed accuracy of 79.7% and AUC of 0.91 [95% CI: 0.85–0.96] in categorization according to BI-RADS. Moderate agreement between dCNN and ground truth has been achieved (*κ*: 0.57 [95% CI: 0.50–0.64]) what is comparable with human readers. Analysis of whole dataset improved categorization accuracy to 90.9% and AUC of 0.91 [95% CI: 0.77–1.00], while achieving almost perfect agreement with ground truth (*κ*: 0.82 [95% CI: 0.69–0.95]), performing on par with human readers. Furthermore, the object localization technique allowed the detection of lesion position slice-wise.

**Conclusions:**

Our results show that a dCNN can be trained to detect and distinguish lesions in ABUS according to the BI-RADS classification with similar accuracy as experienced radiologists.

**Key Points:**

• *A deep convolutional neural network (dCNN) was trained for classification of ABUS lesions according to the BI-RADS atlas*.

• *A sliding-window approach allows accurate automatic detection and classification of lesions in ABUS examinations*.

## Introduction

Breast cancer is one of the most common causes of cancer death in females, and mortality rates are increasing worldwide [1]. In total, 2.3 million women were diagnosed with breast cancer in 2020 alone, causing 685,000 deaths worldwide [2]. The life-time probability to develop breast cancer is estimated to be 12.3% [3] and 1-, 3-, and 5-year survival rates are 92%, 75%, and 73% [4].

Current screening programs most commonly rely on mammography, which is known to reduce breast cancer–related mortality by up to 45% [5]. In spite of its cost-effectiveness, mammography screening exhibits some shortcomings reducing the effectiveness in quality-controlled organized screening programs and opportunistic breast cancer screening. Due to the need of breast compression in conventional mammography, many women decide not to undergo screening mammographies because of the fear of pain [6]. Moreover, dense breast tissue decreases the sensitivity of conventional mammography in cancer detection [7–10]. For women with dense breast tissue or women unwilling to undergo mammography, breast ultrasound may be a robust alternative modality, used as an adjunct to mammography or as an independent screening modality providing an increased accuracy for cancer detection [11, 12] but results are highly dependent on operators’ skill and experience, on top of that requiring an expert’s interpretation of results that may be highly subjective.

The Breast Imaging-Reporting and Data System (BI-RADS) was developed by the American College of Radiology (ACR) as a classification system to standardize quality control and risk assessment in mammography [13, 14]. The BI-RADS guidelines apply a numeric scale to describe the presence of lesions and the probability for malignancy. BI-RADS 1 corresponds to an image with no masses or lesions detected; BI-RADS 2 describes a lesion with no suspicion for malignancy; BI-RADS 3 stands for low probability of malignancy (< 3%); 4a—low suspicion of malignancy (2–10%); 4b—intermediate suspicion (11–50%); 4c—high suspicion (51–94%); and BI-RADS 5 corresponds to high probability of malignancy (> 95%) [15].

Automated breast ultrasound (ABUS) may be used instead of the more commonly applied hand-held ultrasound (HHUS) allowing a standardization of the image acquisition, complete coverage of the whole breast volume, and the delegation of the acquisition task to the technician [16, 17]. In first clinical studies, ABUS demonstrated similar accuracy as hand-held ultrasound; moreover, ABUS increased the cancer detection rate in combination with conventional mammography [18, 19]. One of the disadvantages of ABUS examinations is the large number of images resulting in an increased reading time for inexperienced radiologists. Additionally, a higher reader dependence of ABUS assessments has been reported [20].

Machine learning techniques have been shown to allow detection and classification of breast imaging findings [21], and particularly deep convolutional neural networks (dCNNs) provide a powerful tool in breast imaging, for example in detection and classification of microcalcifications in mammography [22], as well as the detection of breast cancer in hand-held US images and mammographies [23]. Applying dCNNs for feature extraction from ABUS examinations resulted in an increase of diagnostic reliability for second reading [24].

In this study, we trained a dCNN algorithm with ABUS findings to imitate the radiological decision-making according to the ACR BI-RADS recommendations of risk assessment in ABUS examinations. Moreover, a sliding-window approach was implemented for automatic localization of suspicious and non-suspicious lesions in ABUS datasets based on the dCNN classification.

## Materials and methods

### Patient data

This retrospective study has been approved by the local ethics committee (“Kantonale Ethikkommission Zurich”; Approval Number: 2016-00064). ABUS images for training, validation, and testing of the algorithm have been retrospectively gathered from the PACS database of the University Hospital of Zurich among the exams performed between 2017 and 2019. In total, 645 ABUS image sets containing lesions from 113 female patients were selected by an experienced radiologist with over 8 years of experience in breast imaging and over 3 years of experience in ABUS imaging. From the selected data, 55 patients had lesions of high probability for malignancy (BI-RADS 4/5) and 58 patients had lesions of low probability of malignancy (BI-RADS 2/3), all later confirmed as stable lesions or histologically proven fibroadenomas. For training of a model, 189 images containing a lesion with high malignancy probability and 178 images with low probability were used. For model validation, each BI-RADS category contained 75 images. Dataset for testing comprised of 71 images with high malignancy probability from 10 patients and 57 images with low malignancy probability from 10 patients that were not used for training nor validation of a model.

### ABUS examination

Images were acquired with a dedicated ABUS device (Invenia™ Automated Breast Ultrasound System, GE Healthcare) by a trained technician. Acquisition consisted of three volumes per breast in order to cover the whole breast volume. Each image set consists of a stack of 2D images with a matrix size of 600 × 600 pixels; each of them presents a US view plane 0.5 mm apart from each other.

### Image selection and data preparation

**Fig. 1:**
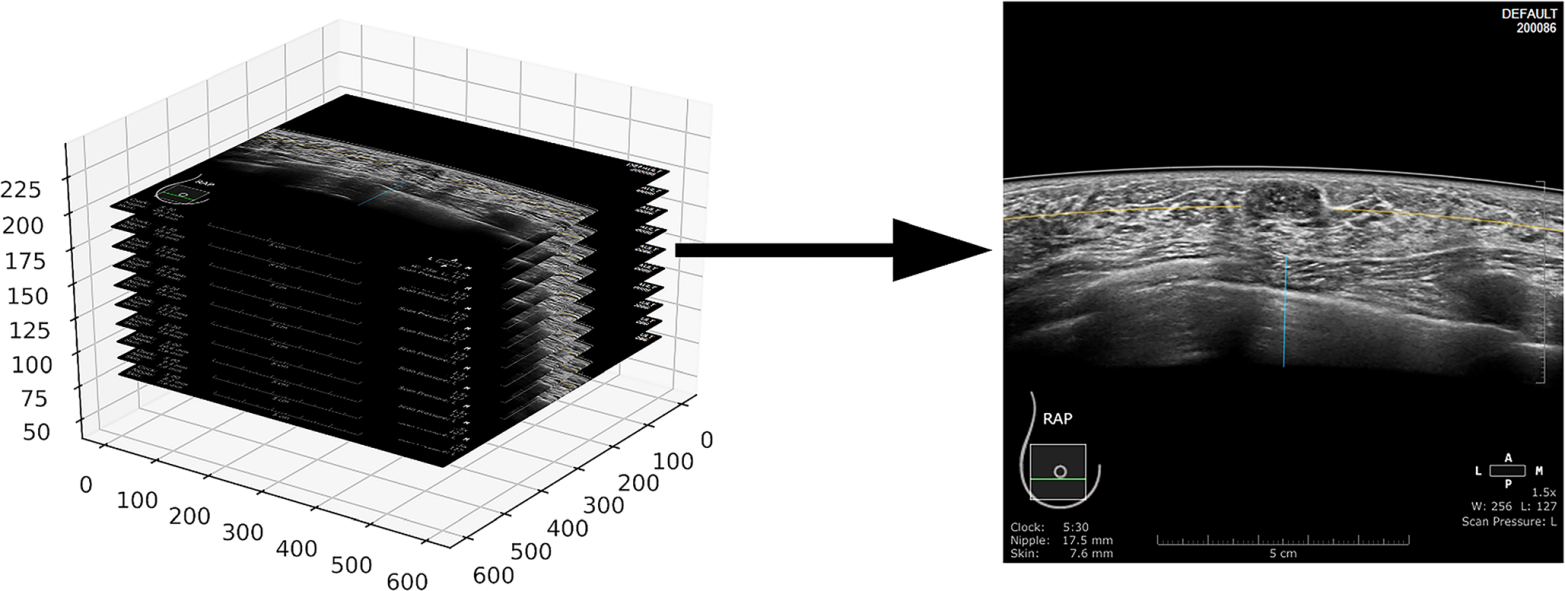
Visualization of single image extraction from ABUS examination dataset

From the complete datasets, single images depicting lesions were selected by the radiologist and classified according to BI-RADS categories. Depiction of a single image from the image stack is shown in Fig. 1. Ten image sets were excluded from further analysis due to presence of artifacts. For training and validation of the dCNN, the area surrounding the lesion was selected by the radiologist and cropped as a rectangular image with a size of 151 × 181 pixels. Training/validation images were augmented to improve the generalization of training and to balance the classes of the dataset using the TensorFlow (Google LLC) data augmentation tool for rotation, height and width shift, shear, zoom, and horizontal flipping. Detailed data on the number of augmented images used for each part of learning process are presented in a Table 1. To allow for a step-wise detection and classification, 3 dCNN models were trained:

- Model 1—distinguishing between image background from breast tissue
- Model 2—distinguishing between normal breast tissue and presence of a lesion (both benign and malignant)
- Model 3—determination of the BI-RADS category of the detected lesion (BI-RADS 2/3 or BI-RADS 4/5)

**Table 1:** Models training and validation dataset information and training and validation results

	Training dataset	Validation dataset	Training accuracy	Validation accuracy
Model 1—Tissues	2560 images	1110 images	99.40%	99.10%
Model 2—Lesions	6241 images	3386 images	98.20%	96.90%
Model 3—BI-RADS 2/3 versus 4/5	7164 images	3530 images	98.50%	98.80%

### dCNN architecture

The models were trained using a deep convolutional neural network with 13 convolutional layers with 5 max pooling layers for downsampling followed by 2 dense layers with the ReLU activation function. A 50% dropout was applied to reduce overfitting and a softmax activation function was used for the final weights. For model training, a stochastic gradient descent optimization was used with binary cross-entropy loss function. Batches of 16 were applied and the learning rate was set to 1.0 × 10^−6^, a Nesterov momentum of 0.90 was used, and training was stopped after training and validation accuracies stopped improving, what meant 120 epochs for models 1 and 2 and 160 epochs for model 3. dCNNs were trained with a TensorFlow 2.0 software library on a machine running Ubuntu 16.04 OS with an Nvidia 1080 GTX GPU. Class weighting was used to reduce the imbalance in training datasets.

### Image analysis and lesion detection

**Fig. 2:**
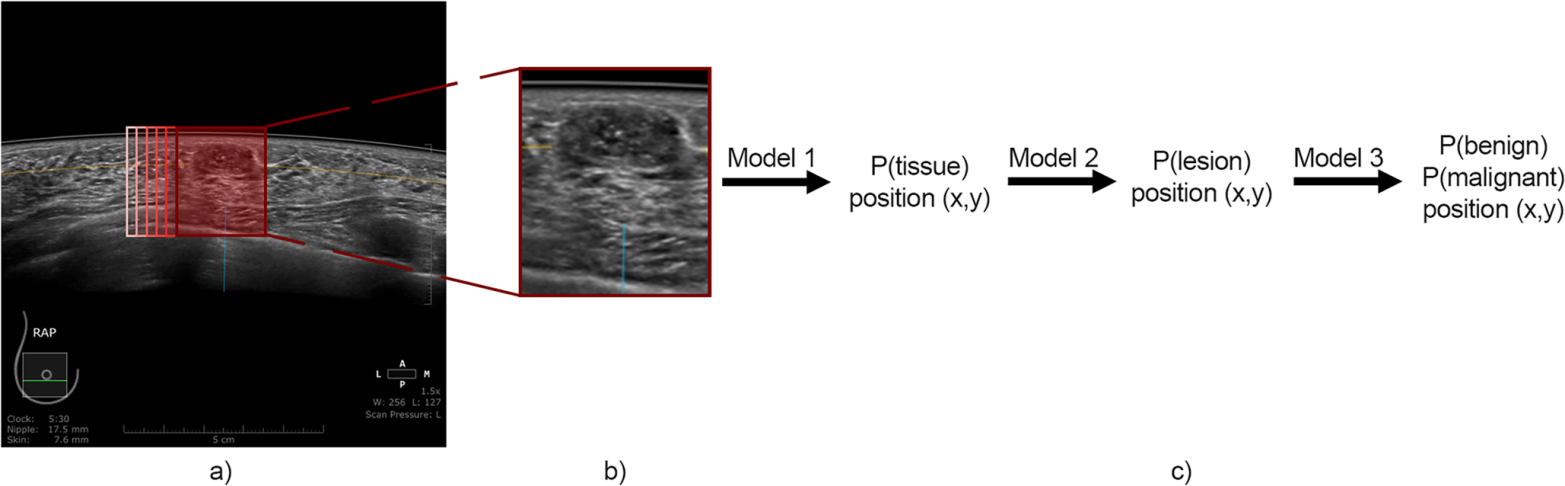
Sliding-window approach for localization and prediction of lesions on images. For analysis of the whole image, separate crops are being selected (**a**). Prediction based on cropped image with a window of a fixed size (**b**). All 3 models are being applied to cropped fragment of an image. Probability result from each of them is written into table with corresponding coordinates of center of an image (**c**)

To localize regions of an image, in which tissue or lesions are depicted, and to create a probability map showing pixel-by-pixel the probability of lesion presence, a sliding-window approach was implemented in the programming language Python 3.5. From the original ABUS image with a size of 600 × 600 pixels, sections of 151 × 181 pixels around given *x*,*y* coordinates were cropped in a loop over *x* and *y*, and with each cropped image the step-wise dCNN classification algorithm was applied. Probability results of calculations were written into 3 arrays (results for each model is written into separate array) with corresponding *x* and *y* coordinates. After all the dCNN evaluations for one cropped area were done, the center on the original image was moved by 10 pixels in *X* or *Y* direction in the loop. A detailed scheme of the sliding-window approach is presented in Fig. 2.

**Fig. 3:**
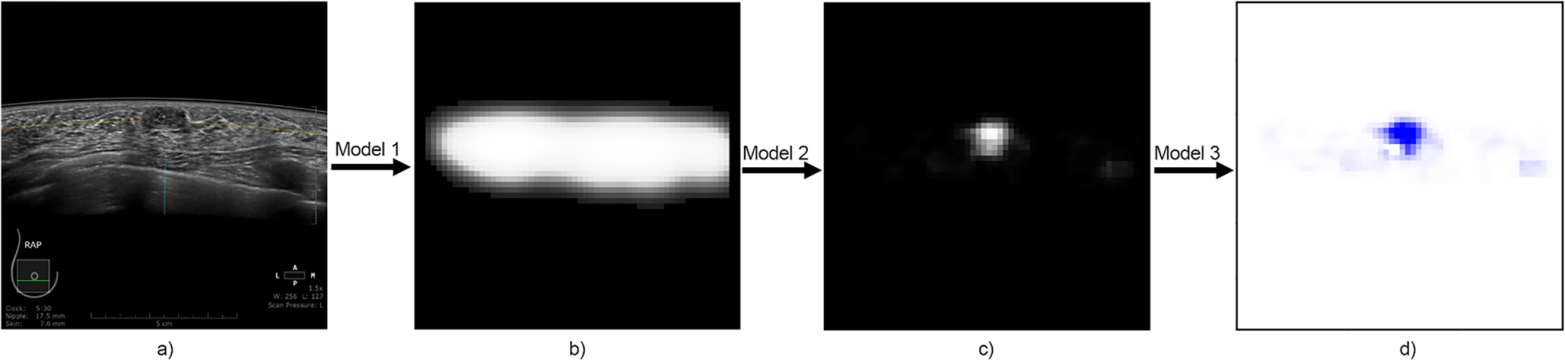
Probability maps are generated for each image. Sliding-window algorithm was applied to the original image (**a**). As a result of model 1 calculations, grayscale probability map was generated presenting area of breast tissue depiction (**b**). Application of model 2 on an image results in depiction of suspected lesion visualized by grayscale probability map (**c**). Lesions were classified according to BI-RADS with model 3 and visualized in blue (BI-RADS 2/3) and red (BI-RADS 4/5) colors (**d**)

For each analyzed image, the sliding-window algorithm results in three 60 × 60 matrices consisting of the class probabilities of the step-wise classification. Results of calculations performed with 3 trained models are visualized as heatmaps giving spatial information on the probability of detection and classification. A visualization of an exemplary calculation is presented in Fig. 3. In the colormaps, results are presented as overlays on the original image in red and blue color representing the probabilities of dCNN classification to the BI-RADS 2/3 or BI-RADS 4/5 categories. Due to the step-wise classification of the image region, only those areas representing a detected lesion (evaluation step 2) were evaluated in step 3 using the model classifying into BI-RADS 2/3 or BI-RADS 4/5 (evaluation step 3). Therefore, only those areas which represent a detected lesion exhibit colors of red or blue in the colormap. To generate probability maps from the complete image volume, the previously described method was consecutively applied to all images from the image stack resulting in a matrix of 60 × 60 × 3 × 330 (3 probability matrices with a dimension 60 × 60 for each of the 330 images from a full examination set).

### Comparison with human reading

The test dataset was categorized into the three categories BI-RADS 2 (no suspicion of breast cancer), BI-RADS 3 (low probability of breast cancer), and BIRADS 4/5 (high likelihood of breast cancer) by two radiologists, reader 1 with more than 10 years of experience in breast imaging and reader 2 with more than 5 years of experience in breast imaging. All lesions were also classified according to the previously described 3 categories by the step-wise dCNN algorithm for lesion detection and classification.

**Fig. 4:**
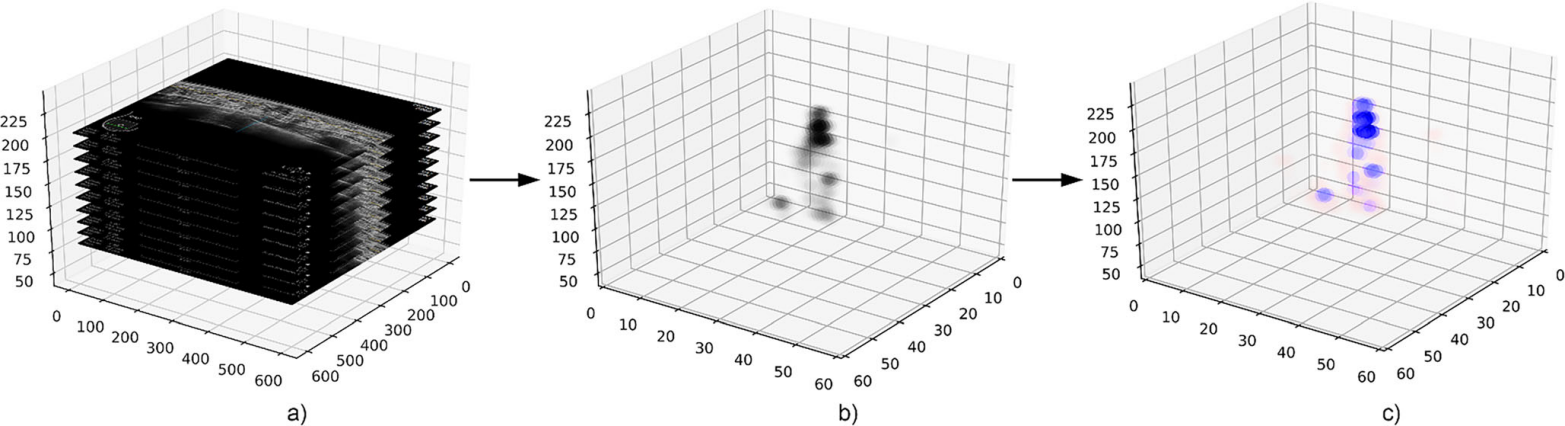
Presentation of probability results in 3D space. In a whole ABUS dataset (**a**), each image underwent the prediction algorithm. Based on slice spacing, result of lesion localization is plotted in 3D space (**b**) and probability of malignancy is evaluated and plotted in 3D (**c**)

Intra-reader reliability was obtained by a repeated categorization of the same datasets by the same radiologists after a period of 3 months (Fig. 4).

### Statistical analysis

The statistical analysis was performed using the Scikit-learn 0.22.1 package for the Python programming language. Confusion matrices were applied to compare the performance of the dCNN with the ground truth categorization (human readers). Agreement of categorization between dCNN and human readers and inter- and intra-reader correlations have been calculated using Cohen’s kappa coefficient (*κ*). The diagnostic performances of the trained models were calculated using receiver operating characteristics. Diagnostic accuracy was evaluated in a form of binary classification accuracy and area under the curve (AUC) statistic of ROC curves with a confidence interval *CI* = 0.95 from Monte Carlo simulation.

## Results

### Model training

**Fig. 5:**
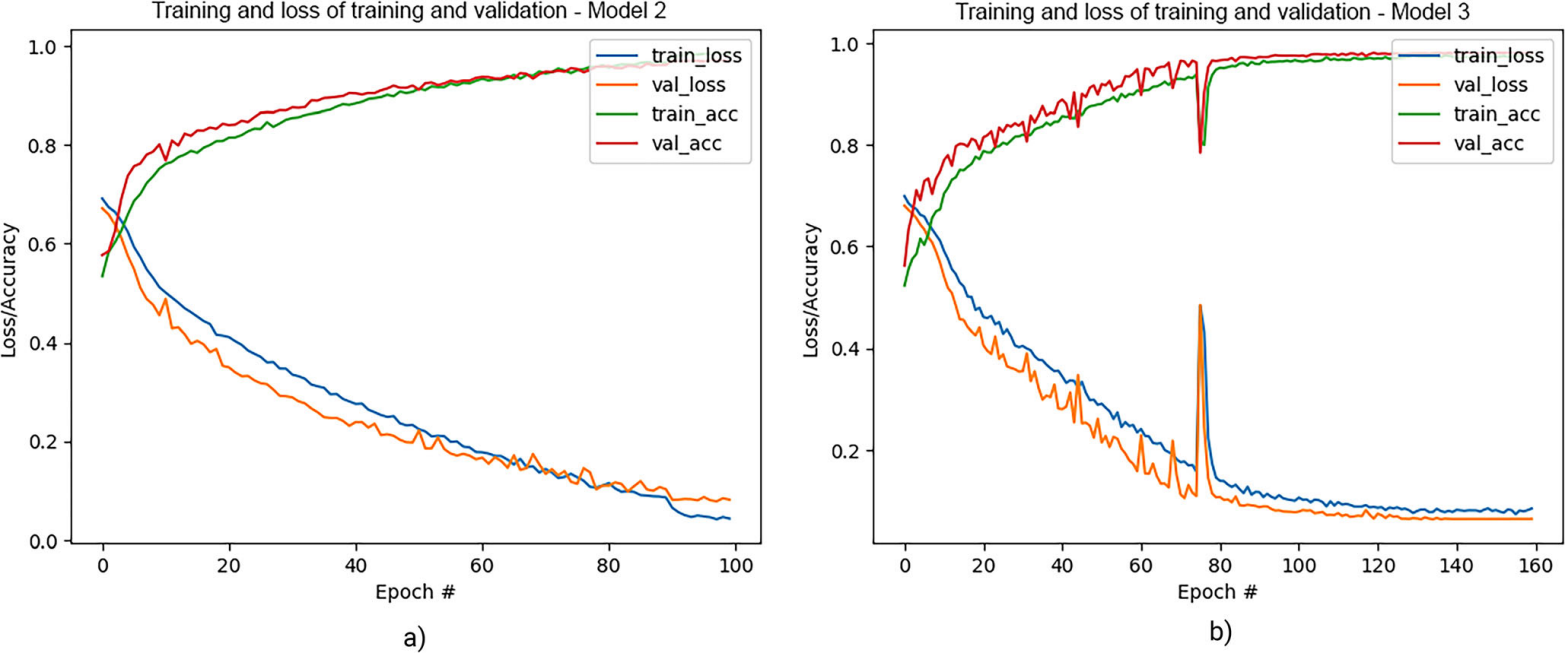
Results of training and validation of model 2 (**a**) and model 3—BI-RADS categorization (**b**)

Training of the dCNN models resulted in accuracies between 98.2 and 99.0% on the training datasets and between 96.9 and 99.1% accuracy on the validation datasets. Training and validation curves for the model distinguishing between normal tissue and lesions (model 2) and for the model classifying a detected lesion in probably benign (BI-RADS 2/3) or suspicious (BI-RADS 4/5) are presented in Fig. 5. Detailed results of the training and validation accuracies and the number of images are presented in Table 1.

### Validation on single images presenting lesions

For testing the dCNN models with data not used for training or validation, a dataset of 128 images was applied, each depicting a lesion of either BI-RADS 2/3 or BI-RADS 4/5, using the double-read radiological report from the RIS archive as ground truth. In the image analysis, the dCNN and two experienced human readers were compared treating each image as an independent case.

**Fig. 6:**
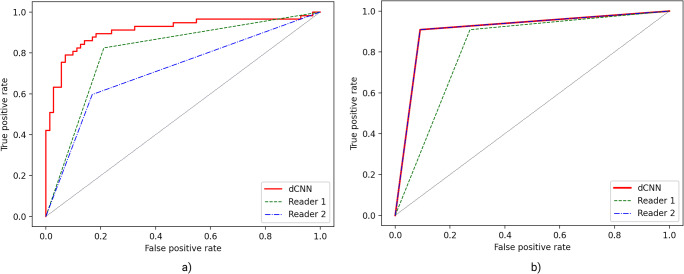
ROC curves for dCNN and both readers showing discrimination capabilities of test dataset based on single images (**a**) and discrimination capabilities of test dataset based on full examination imageset (**b**)

Using model 3 for analysis, an accuracy of 79.7% was found, and a ROC analysis for model 3 leads to an AUC of 0.91 (AUC: 0.91 [95% CI: 0.85–0.96]) with Youden’s index *J* of 0.92. AUC for reader 1 was 0.80 [95% CI: 0.73–0.87], and for reader 2 AUC was 0.71 [95% CI: 0.63–0.78]. Confusion matrices for the dCNN model and both human readers are presented in Table 2, detailed results regarding accuracies, inter-reader agreement, and ROC analyses are presented in Table 3 and Table 4, and corresponding ROC curves are depicted in Fig. 6.

**Table 2:**
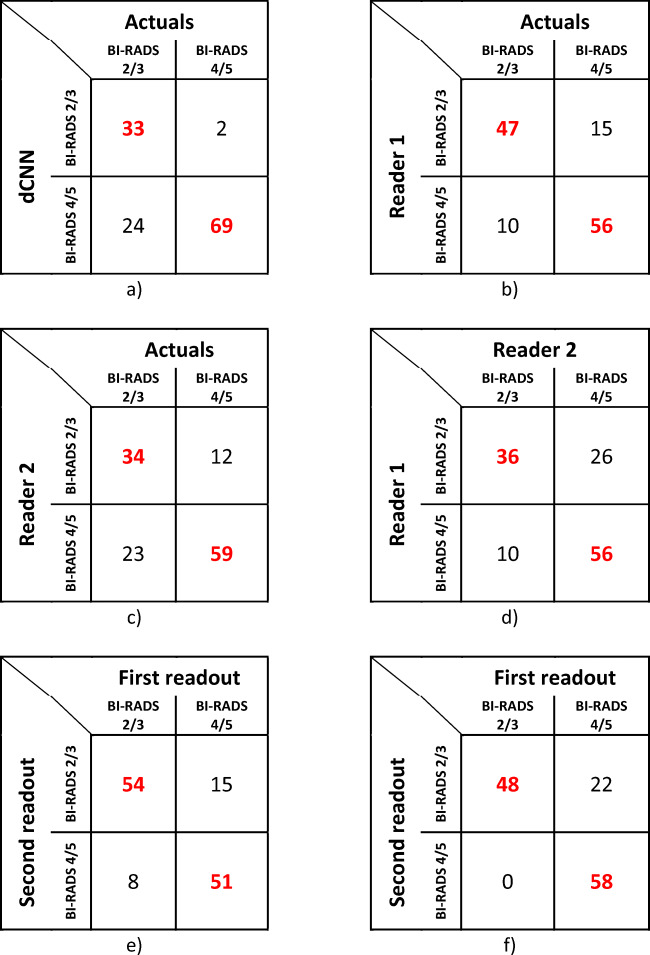
Confusion matrices comparing BI-RADS 2/3 and BI-RADS 4/5 classification results of dCNN (**a**), human reader 1 (**b**), and reader 2 (**c**) to ground truth (actuals) based on single images and inter-reader agreement (**d**), intra-reader reliability for reader 1 (**e**) and reader 2 (**f**)

**Table 3:** Inter-rater agreement for both image-wise and for full examination for both readers and the dCNN compared to ground truth

		**Accuracy [%]**	***κ*** **(95% CI)**	**Agreement**
**Single images**	**dCNN/actuals**	79.7%	0.57 (0.50–0.64)	Moderate
	**Reader 1/actuals**	80.5%	0.61 (0.53–0.68)	Substantial
	**Reader 2/actuals**	72.7%	0.44 (0.36–0.51)	Moderate
	**Reader 1/Reader 2**	71.9%	0.43 (0.28–0.58)	Moderate
**Full imageset**	**dCNN/actuals**	90.9%	0.82 (0.69–0.95)	Almost perfect
	**Reader 1/actuals**	81.8%	0.63 (0.47–0.81)	Substantial
	**Reader 2/actuals**	90.9%	0.82 (0.69–0.95)	Almost perfect
	**Reader 1/Reader 2**	72.7%	0.46 (0.09–0.82)	Moderate

**Table 4:** ROC analysis for the test dataset in relation to radiological report as ground truth

		**AUC (95% CI)**	**Specificity [%]**	**Sensitivity [%]**	**PPV [%]**	**NPV [%]**
**Single images**	**Reader 1**	0.80 (0.73–0.87)	78.9%	82.5%	75.8%	84.9%
	**Reader 2**	0.71 (0.63–0.78)	83.1%	59.7%	73.9%	72.0%
	**dCNN**	0.91 (0.85–0.96)	97.2%	57.9%	94.3%	74.2%
**Full imageset**	**Reader 1**	0.82 (0.68–1.00)	72.7%	90.9%	76.9%	88.9%
	**Reader 2**	0.91 (0.77–1.00)	90.9%	90.9%	90.9%	90.9%
	**dCNN**	0.91 (0.77–1.00)	90.9%	90.9%	90.9%	90.9%

The agreement between the dCNN model 3 and the ground truth was moderate (*κ*: 0.57 [95% CI: 0.50–0.64]). The agreement between reader 1 and the ground truth was substantial (*κ*: 0.61 [95% CI: 0.53–0.68]), whereas the agreement between reader 2 and the ground truth was moderate (*κ*: 0.44 [95% CI: 0.36–0.51]). Agreement between readers was moderate (*κ*: 0.43 [95% CI: 0.28–0.58]).

Intra-reader agreement was substantial for both reader 1 (*κ*: 0.64 [95% CI: 0.54–0.77]) and reader 2 (*κ*: 0.66 [95% CI: 0.54–0.78]).

### Validation on full 3D ABUS datasets

Analyzing complete 3D ABUS datasets and classifying this dataset according to the highest BI-RADS score of lesions present, the accuracy of classification of the algorithm improved to 90.9% compared to the radiological report as a ground truth. ROC analyses for dCNN resulted in an AUC of 0.91 (AUC: 0.91 [95% CI: 0.77–1.00]) with Youden’s index *J* of 0.92. The AUC for reader 1 was 0.82 [95% CI: 0.68–1.00] and for reader 2 was 0.91 [95% CI: 0.77–1.00]. ROC curves for the analysis of complete ABUS datasets are depicted in Fig. 6, and the confusion matrices are shown in Table 5.

**Table 5:**
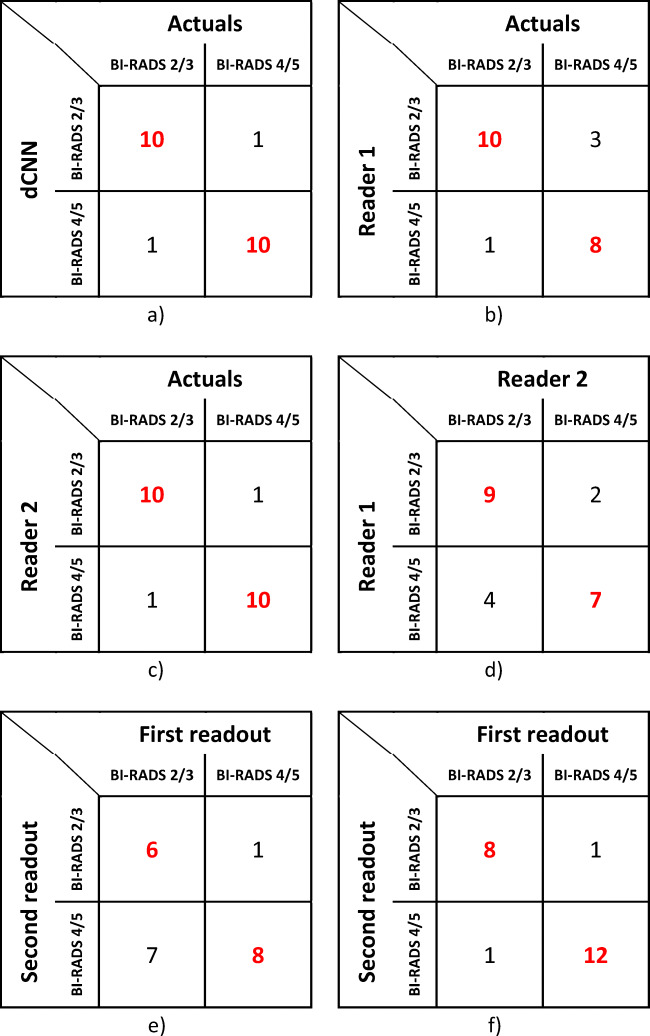
Confusion matrices comparing BI-RADS 2/3 and BI-RADS 4/5 classification results of dCNN (**a**), human reader 1 (**b**), and reader 2 (**c**) to ground truth (actuals) based on full examination imageset and inter-reader agreement (**d**), intra-reader reliability for reader 1 (**e**) and reader 2 (**f**)

The agreement between the dCNN algorithm and the ground truth was almost perfect (*κ*: 0.82 [95% CI: 0.60–0.95]) and the agreement between readers and ground truth was substantial to almost perfect (reader 1—*κ*: 0.63 [95% CI: 0.47–0.81] and reader 2—*κ*: 0.82 [95% CI: 0.60–0.95]). Agreement between readers was moderate (*κ*: 0.46 [95% CI: 0.09–0.82]).

Intra-reader agreement was fair (*κ*: 0.32 [95% CI: 0.01–0.65]) for reader 1 and almost perfect (*κ*: 0.81 [95% CI: 0.56–1.00]) for reader 2.

## Discussion

In the current study, we were able to show that a deep convolutional neural network (dCNN) can be trained to classify lesions in ABUS according to the ACR BI-RADS classification with accuracies higher than 95% in both the training and the validation datasets. Compared to human reading by experienced radiologists, the dCNN showed similar diagnostic accuracy on test datasets not used for training of the machine learning algorithm, and also inter-reader agreement between the dCNN was comparable to the inter-reader agreement of experienced radiologists. Applying a 3-dimensional sliding-window approach with a step-wise assessment of the region of interest, the dCNN was able to detect and classify lesions according to the BI-RADS atlas with accuracy providing a technique for the automatic reading of 3D ABUS datasets mimicking the human workflow.

In the clinical routine, the evaluation of radiological breast images is standardized according to the BI-RADS atlas grading the probability of the presence of breast cancer based on atlas images. In spite of this standardization, the quality of radiological decision-making is dependent on the experience of the reader as well as the workload. It has been shown that even after short-term re-evaluation of images, up to 29% of BI-RADS assessments may be reclassified [25]. The usability of AI models as a support tool for radiologists in the decision-making process has been shown in several studies, e.g. [26], which particularly can be used to decrease the false-positive rates in mammography screening [27].

Ultrasound as an adjunct to mammography has been shown to decrease the false-negative rate of conventional mammography, and ABUS may be used instead of hand-held ultrasound exhibiting similar cancer detection rates [28]. As the ABUS examination is typically carried out by the technician, a workflow improvement is achieved in radiological institutions replacing hand-held ultrasound with ABUS. Moreover, as the complete breast volume is examined and stored as 3D datasets, ABUS is a step further in the standardization of breast imaging workflow. Another unwanted effect of ABUS is the relatively long reading time and the higher rate of false-negative lesions. Here, our proposed algorithm may offer a significant improvement: it highlights suspicious lesions in the large volumetric 3D dataset and gives also a suggestion of the correct classification of the lesion, which may be helpful for inexperienced readers. Application of AI in clinical routine potentially offers a reliable second opinion tool for experienced radiologists as well as providing a training platform for inexperienced readers. An AI solution is observer-independent and minimizes the impact of experience and external factors for diagnosis. Instead of working as a “black-box,” the explainable AI concept is applied in this study. By providing step-wise solutions, each decision made by the AI program can be traced back, which enables deeper understanding and adaptation of the AI program in clinical use.

The accuracy of our algorithm trained for classification of ABUS lesions is comparable to the results of Wang et al [24], who used multiview CNNs to classify breast lesions in datasets obtained from a breast volume scanner with accuracy in the order of 88%. Lee et al showed that similar algorithms allow for precise lesion segmentation with Spearman’s correlation coefficient of *ρ* = 0.929 [29].

In clinical usage, CAD software has proven to significantly improve and accelerate workflow. Those systems have already proven its high sensitivity in mammography with detection of masses of 91% on initial and 89% on follow-up mammograms [30] as well as ultrasonography with sensitivity of 90.9% [31]. ABUS’s main advantages over other examination types, like volumetric representation and relative procedure simplicity, increase requirements for results analysis for radiologists. CAD systems allow to overcome this issue and provide reliable second opinion to specialist [32]. The application of CAD software for ABUS in clinical use demonstrated an evaluation speed up by 9–15% [33, 34]. Segmentation methods used for CAD have shown to improve the ability to distinguish positive from negative cases (AUC) by 6% when used as support program by inexperienced readers [35]. Commercially available AI CAD software based on minimum intensity projection visualization (MinIP) improves sensitivity of the readout by 5.2–10.6%, but may come with a possible decrease of specificity in the range of 1.4–5.7%, while additionally requiring parameter adjustment for readout that affects sensitivity [36]. Even though proven to work in a clinical environment, those methods come with limitations in regard to irregularities and various lesion sizes what may lead to an increased number of false-positives [37] and may require parameter fine-tuning and extensive image preprocessing [38]. The presented method provides improved generalization and allows a possibility for customization for specific manufacturers and adjustment to internal work processes.

Our study has some limitations: (1) A retrospective study design was chosen for validating the algorithm resulting in potential bias due to the choice of datasets. However, a prospective evaluation was out of the scope of this methodological study. (2) All data was gathered from one single institution and data from only one device supplier was used for acquisition. Nonetheless, the purpose of this study was not to demonstrate that our algorithm works well on every possible clinical setup but to demonstrate in principle that an algorithm can be trained to mimic human reading in ABUS examinations. (3) Although data was derived from the general female patient cohort of our institution, not all ethnicities were included in this study. (4) Only one dCNN architecture and detection algorithm has been investigated and no comparison with pretrained models has been made; therefore, we cannot exclude that other dCNN architectures potentially would result in even higher accuracies.

In conclusion, the trained AI models allowed for further standardization as an independent second opinion tool in ABUS examinations according to BI-RADS catalogue. Implementation of our AI models into the clinical routine potentially could improve reliability of assessment while reducing workload for radiologists.

## Abbreviations

ABUS: Automated breast ultrasound
ACR: American College of Radiology
AUC: Area under the curve
BI-RADS: Breast Imaging-Reporting and Data System
CAD: Computer-aided detection
dCNN: Deep convolutional neural network
HHUS: Hand-held ultrasound
ROC: Receiver operating characteristic
US: Ultrasound

## Declarations

### Guarantor

The scientific guarantor of this publication is Andreas Boss, MD PhD.

### Statistics and biometry

No complex statistical methods were necessary for this paper.

### Methodology

• retrospective

• diagnostic or prognostic study

• performed at one institution
